# A systematic scoping review moral distress amongst medical students

**DOI:** 10.1186/s12909-022-03515-3

**Published:** 2022-06-17

**Authors:** Rui Song Ryan Ong, Ruth Si Man Wong, Ryan Choon Hoe Chee, Chrystie Wan Ning Quek, Neha Burla, Caitlin Yuen Ling Loh, Yu An Wong, Amanda Kay-Lyn Chok, Andrea York Tiang Teo, Aiswarya Panda, Sarah Wye Kit Chan, Grace Shen Shen, Ning Teoh, Annelissa Mien Chew Chin, Lalit Kumar Radha Krishna

**Affiliations:** 1 Yong Loo Lin School of Medicine, National University of Singapore, NUHS Tower Block, 1E Kent Ridge Road, Level 11, 119228 Singapore, Singapore; 2 Division of Supportive and Palliative Care, National Cancer Centre Singapore, 11 Hospital Dr, 169610 Singapore, Singapore; 3 Medical Library, National University of Singapore libraries, Singapore Blk MD6, Centre, 14 Medical Dr, #05-01 for Translational Medicine, Singapore, Singapore; 4 Division of Cancer Education, National Cancer Centre Singapore, 11 Hospital Dr, Singapore, 169610 Singapore; 5 Duke-NUS Medical School, National University of Singapore, Singapore 8 College Rd,, Singapore, 169857 Singapore; 6 Palliative Care Institute Liverpool, Academic Palliative and End of Life Care Centre, Cancer Research Centre, University of Liverpool, 200 London Rd, Liverpool, L3 9TA UK; 7 Centre of Biomedical Ethics, National University of Singapore, 21 Lower Kent Ridge Rd, Singapore, 119077 Singapore; 8 PalC, The Palliative Care Centre for Excellence in Research and Education, PalC c/o Dover Park Hospice, 10 Jalan Tan Tock Seng, Singapore, 308436 Singapore

**Keywords:** Medical students, Moral distress, Ring Theory of Personhood (RToP), Personhood

## Abstract

**Background:**

Characterised by feelings of helplessness in the face of clinical, organization and societal demands, medical students are especially prone to moral distress (MD). Despite risks of disillusionment and burnout, efforts to support them have been limited by a dearth of data and understanding of MD in medical students. Yet, new data on how healthcare professionals confront difficult care situations suggest that MD could be better understood through the lens of the Ring Theory of Personhood (RToP). A systematic scoping review (SSR) guided by the RToP is proposed to evaluate the present understanding of MD amongst medical students.

**Methods:**

The Systematic Evidence-Based Approach (SEBA) is adopted to map prevailing accounts of MD in medical students. To enhance the transparency and reproducibility, the SEBA methodology employs a structured search approach, concurrent and independent thematic analysis and directed content analysis (Split Approach), the Jigsaw Perspective that combines complementary themes and categories, and the Funnelling Process that compares the results of the Jigsaw Perspective with tabulated summaries to ensure the accountability of these findings. The domains created guide the discussion.

**Results:**

Two thousand six hundred seventy-one abstracts were identified from eight databases, 316 articles were reviewed, and 20 articles were included. The four domains identified include definitions, sources, recognition and, interventions for MD.

**Conclusions:**

MD in medical students may be explained as conflicts between the values, duties, and principles contained within the different aspects of their identity. These conflicts which are characterised as disharmony (within) and dyssynchrony (between) the rings of RToP underline the need for personalised and longitudinal evaluations and support of medical students throughout their training. This longitudinal oversight and support should be supported by the host organization that must also ensure access to trained faculty, a nurturing and safe environment for medical students to facilitate speak-up culture, anonymous reporting, feedback opportunities and supplementing positive role modelling and mentoring within the training program.

**Supplementary Information:**

The online version contains supplementary material available at 10.1186/s12909-022-03515-3.

## Background

Moral distress (MD) amongst healthcare professionals (HCP)s is an increasing concern [1–3] amidst the COVID-19 pandemic [4–6]. Characterised by “*a lack of assertiveness or autonomy, socialization pressures to follow others, lack of time, inhibiting power structure, lack of collegial support, and organizational priorities that conflict with care needs*” ([7] p. 4), medical students are seen to be especially prone to MD. These concerns are further heightened by data suggesting that poor ethical climes, uncooperative environments, and pressure to carry out or make allowances for unethical acts ([8–15] #95) that predispose to MD have been identified in educational settings involving medical students [16–18]. This underscores the need to better understand the effects of MD on medical students and to ensure effective support of ‘at risk’ medical students [19–21].

However, a dearth of knowledge on MD amongst medical students and the notion that MD is a personalised sociocultural construct, that is the product of the tension between an individual’s ethical, moral, relational, situational, personal, professional, and societal values, beliefs and principles and regnant sociocultural, institutional and professional expectations, standards and codes of practice, emphasises the need to review how current understanding of MD is mapped [12–14, 22].

Drawing upon insights into how HCPs cope with grief and bereavement [15], address complicated care issues [5, 19–21, 23, 24] and care for dying patients [23, 25–36] that result in similar conflicts between deeply held beliefs, principles, values, and prevailing roles, expectations, and responsibilities and that manifest emotions and reactions akin to that described in MD, we posit that MD may also be better understood through the lens of personhood [37–39]. Kuek, Ngiam [25], Ho, Kow [30], Ngiam, Ong [34], Chan, Chia [31] and Huang, Toh [26] review how nurses, doctors and medical students face the deaths of their patients and cope with caring for the dying suggest that the Ring Theory of Personhood (RToP)’s is well equipped to map conflicts between deeply held beliefs, principles, values, and prevailing roles, expectations, and responsibilities that mirror those seen in [5, 23, 24–40] MD [5, 8–11, 15, 19–21, 23–40].

### The Ring Theory of Personhood (RToP)

Kuek, Ngiam [29] Kuek, Ngiam [29] Ho, Kow [34] Ngiam, Ong [38]Chan, Chia [35] Ngiam, Ong [38]Chan, Chia [35] Huang, Toh [23, 41–43] Kuek, Ngiam [29] Radha Krishna and Alsuwaigh [31]Kuek, Ngiam [25] suggests Radha Krishna and Alsuwaigh [28]’s Ring Theory of Personhood (RToP), [41–43]could help identify medical students facing or at risk of MD and direct timely, holistic, personalised, and appropriate support that will attenuate the risk of burnout, attrition in the profession and compromised patient care [12–14, 22].

**Fig. 1:**
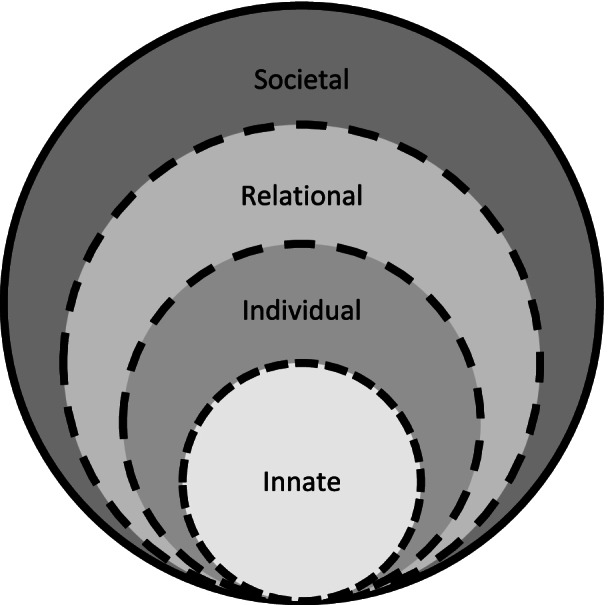
The Ring Theory of Personhood (RtoP) [28]

The RToP captures concepts of personhood – or “what makes you, you” [28] and is depicted by 4 rings depicting the (1) Innate, (2) Individual, (3) Relational, and the (4) Societal Rings (Fig. 1).

At the core of the Ring Theory is the Innate Ring. Krishna and Alsuwaigh defined the Innate Ring as containing the rights and privileges bestowed to all persons by virtue of their being living humans, conceived due to their connections with the Divine or their genetic makeup [23, 28, 29]. Innate Ring also houses the individual’s spiritual, religious and/or theist beliefs and values which are shaped by the individual’s demographical and historical features such as the ethnicity, culture, religion, family unit, gender, society, country, and the social group they were born into [23, 28, 29]. These individualized beliefs, values, moral ideals, and ethical principles influence the individual’s thinking, goals, motivations, and actions, which are expressed through the Individual Ring [23, 28, 29].

The Individual Ring encompasses and builds upon the Innate Ring and represents a conscious function which includes the ability to think, feel, communicate, carry out actions, and interact with the environment [23, 28, 29]. The Individual Ring confers a sense of individuality shaped by the individual’s values, beliefs, principles, biases, preferences, thoughts, emotions, experiences, decision making and personality drawn from the values, beliefs and principles contained in the other three rings [23, 28, 29].

The Relational Ring consists of personal relationships deemed important to the individual, such as family members, loved ones and close friends [23,28, 29]. These ties are determined by the person and can change over time [23, 28, 29].

The Societal Ring is the outermost ring that consists of less intimate relationships such as those shared with colleagues and acquaintances. The Societal Ring also contains societal, religious, professional, and legal expectations set out in the individual’s society to guide and police conduct [23, 28, 29].

Critically each ring also represents an element of the medical student’s identity and the values, beliefs and principles associated with it [23, 28–31]. This link between personhood and identity affords the RToP a key role in this review. The Innate Identity drawn from the Innate Ring considers religious, gender, cultural, community-based beliefs, moral values and ethical principles. The Individual Identity encompasses personal values, beliefs, and personalities whilst the Relational and Societal Identities drawn from the outermost rings pivot on familial and societal values, beliefs, expectations, and principles, respectively [23, 28, 29, 31] Kuek, Ngiam [29, 31].

Chan, Chia [31] Kuek, Ngiam [25], and Ngiam, Ong [38]Chan, Chia [35] Ngiam, Ong [34] suggest that when the beliefs, moral values, ethical principles, familial mores, cultural norms, attitudes, thoughts, decisional preferences, roles, and responsibilities housed in each of these rings come into conflict in a variety of situations, disharmony and dyssynchrony arise. Disharmony refers to conflicts between values, beliefs, and principles *within* the rings whilst dyssynchrony refers to conflicts *between* the rings [28]. It is posited that unresolved disharmony and or dyssynchrony results in MD [25]. These considerations further explain our use of the RToP to guide this review.

### Methodology

Krishna’s Systematic Evidence-Based Approach (henceforth SEBA) [40] is employed to structure a systematic scoping review (henceforth SSR in SEBA) of accounts of MD amongst medical students. To enhance accountability and transparency the SSRs in SEBA employ an expert team to guide, oversee and support all stages of SEBA. In this case, the expert team is composed of medical librarians from the Yong Loo Lin School of Medicine (YLLSoM) at the National University of Singapore and the National Cancer Centre Singapore (NCCS), and local education experts and clinicians at NCCS, the Palliative Care Institute Liverpool, YLLSoM and Duke-NUS Medical School, henceforth the expert team. The expert team enhances the reflexivity of the review. The research team also maintained a reflexive diary to highlight their biases, positions, and assumptions.

SSRs in SEBA are built on a constructivist perspective which acknowledges MD as a sociocultural construct informed by prevailing clinical, academic, personal, research, professional, ethical, psychosocial, emotional, legal and educational factors, the individual’s particular circumstances, their self-concept of personhood and the support available to them at the time [41–45]. SEBA’s relativist lens considers various perspectives through data collected from quantitative, qualitative and knowledge synthesis articles.

**Fig. 2:**
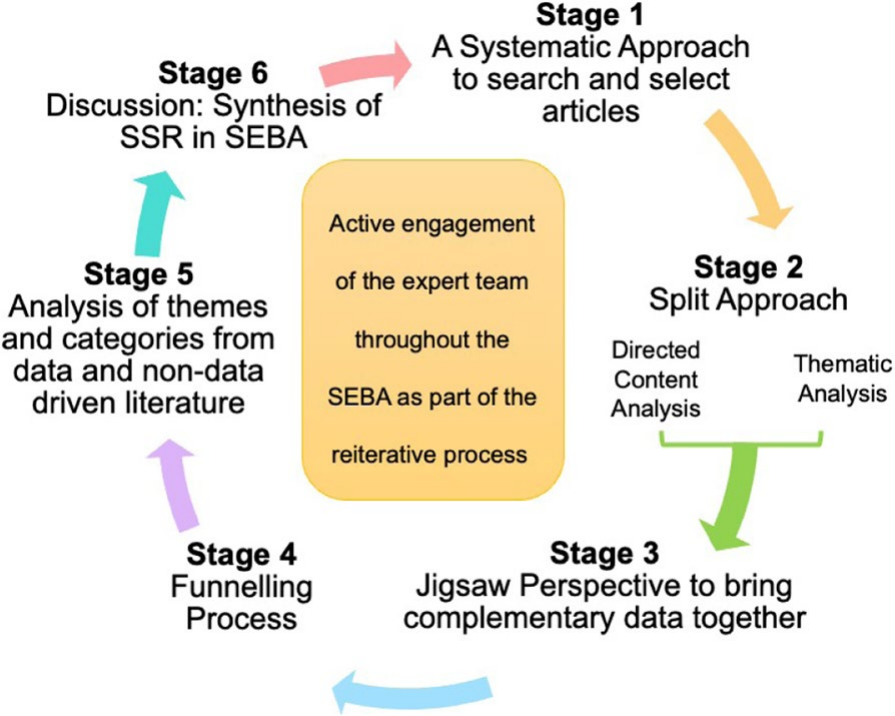
The SEBA process

To operationalise an SSR in SEBA the research team adopted the principles of interpretivist analysis, to enhance reflexivity and discussions [46–49] in the Systematic Approach, Split Approach, Jigsaw Perspective, Funnelling Process, analysis of data from the grey and black literature and Synthesis of SSR in SEBA which make up SEBA’s 6 stages outlined in Fig. 2.

### Stage 1 of SEBA: systematic approach

#### Theoretical lens

The use of the RToP as a theoretical lens is consistent with the Social Cognitive Theory’s posit of a “*triadic reciprocal dynamic relationship between the learner, the environment and the behaviour itself*” [50]. The RToP provides a sketch of the conflicts between a medical student’s beliefs, moral values, ethical principles, familial mores, cultural norms, attitudes, thoughts, decisional preferences, roles, and responsibilities (henceforth values, beliefs, and thoughts) within the 4 aspects of the medical student’s identity. The RToP also offers a better understanding of their contextual and environmental factors, enhancing understanding of their motivations, cognition, responses, thoughts, motivations, biases, ideas, choices, actions, and goals providing a holistic appreciation of the conflicts that underpin MD [23, 25–31, 51]

#### Determining the title and research question

To ensure a systematic approach, the research and expert teams established the goals of the SSR and the population, context, and concept (PCC) to be evaluated. The primary research question was determined to be: “*What is known about MD amongst medical students*?” and the secondary questions were: “*What are the sources of MD in medical students*?” and *“What are the interventions employed to help medical students cope with MD*?”

#### Inclusion criteria

A PICOS format was adopted to guide the research process as shown in Table 1 [52, 53].

**Table 1:** PICOS, inclusion criteria and exclusion criteria applied to literature search

	Inclusion Criteria	Exclusion Criteria
Population	All undergraduate medical students	Papers with the focus on other healthcare students or students from other faculties • Nursing students • Allied health students (e.g. Pharmacy, Psychology, Dietetics, Chiropractic, Midwifery, Podiatry, Social Work, Speech Therapy, Occupational and Physiotherapy) • Non-medical students (e.g. Veterinary, Dentistry, Clinical and Translational Science, Alternative and Traditional medicine) Papers with the main focus on only general physicians, caregiver, family, and patients
Interest	Having moral distress (MD) • Moral distress and ethical distress are both referred to the psychological response when there is the inability to do the right thing. They are used interchangeably in literature and have the same meaning • Moral distress is (a) the psychological distress of (b) being in a situation in which one is constrained from acting (c) on what one knows to be right. • Fourie, 2013: specific psychological response to morally challenging situations such as those of moral constraint or moral conflict, or both	Not faced with any morally challenging situations
Context	Healthcare setting • Including but not limited to acute hospitals, intensive care units, community hospitals, nursing homes and clinics Education setting • During formal and informal curriculum, clinical postings, interaction with stakeholders, educators, peers, other healthcare professionals	Home setting • Personal interactions with family and friends Interactions with members of the public outside clinical and educational settings
Outcome		
Study design	All study designs including: • Mixed methods research, meta-analyses, systematic reviews, randomized controlled trials, cohort studies, case-control studies, cross-sectional studies, descriptive papers, grey literature, opinions, letters, commentaries and editorials Articles in English or translated to English Year of publication: 1 January 1990 to 31 December 2021	

#### Searching

The six members of the research team carried out independent searches of seven bibliographic databases (PubMed, Embase, PsycINFO, ERIC, SCOPUS, Web of Science, Google Scholar) for articles published between 1st January 1990 and 31st December 2021. The searches were carried out between 13th February 2021 and 5th May 2021 and between 17th December 2021 and 17th January 2022. The PubMed search strategy may be found in Additional file 1: Appendix A.

Each member of the research team independently sieved through all titles and abstracts from the individual searches of the four databases and created their own lists of titles to be reviewed. Comparing these individual lists via online meetings, the teams used ‘negotiated consensual validation’ to achieve consensus on the final list of titles to be reviewed [54, 55].

The research team then independently reviewed each of the full-text articles from this final list, created individual lists of articles to be included, discussed these online and achieved a consensus on the final list of full-text articles to be included in the SSR. The results of this process are outlined below.

#### Assessing the quality of included articles

Three research team members individually appraised the quality of the quantitative and qualitative studies using the Medical Education Research Study Quality Instrument (MERSQI) [56] and the Consolidated Criteria for Reporting Qualitative Studies (COREQ) [51, 57]. The MERSQI tool had the following domains: study design, sampling, type of data, validity of evaluation instrument, data analysis and outcomes. The COREQ tool had the following domains: research team and reflexivity, study design, analysis and findings. This allowed the research team to evaluate the methodology employed in the included articles, aid readers and reviewers in appraising the weight afforded the data in the analysis and assist decision-makers in understanding the transferability of the findings. No articles were excluded based on the results of the appraisal.

### Stage 2 of SEBA: split approach

Three teams of at least three researchers independently reviewed the included full-text articles. Wong, Greenhalgh [58] Popay, Roberts [59] The first team summarized and tabulated them in keeping with Wong, Greenhalgh [60]’s RAMESES publication standards: meta-narrative reviews and Popay, Roberts [61]’s “Guidance on the conduct of narrative synthesis in systematic reviews”. The tabulated summaries ensure that key points of the articles are not lost (Additional file 2: Appendix B).

Concurrently, the second team independently analysed the included articles using Braun and Clarke [62] Braun and Clarke’s approach to thematic analysis while the third team adopted Hsieh and Shannon’s Hsieh and Shannon [63] approach to directed content analysis. Radha Krishna and Alsuwaigh [31] Concurrent use of thematic and directed content analysis is a key feature of the ‘Split Approach’ and serves to enhance the reproducibility, transparency, and accountability of the analytic process. This concurrent analysis also serves to reduce the omission of new findings or negative reports and enable review of data from different perspectives.

#### Thematic analysis

In the absence of rigorous definitions of MD, three members of the research team adopted Braun and Clarke’s approach to identify key themes across different learning settings and medical student populations. This allowed for the analysis of data derived from quantitative, qualitative, and mixed methodologies. This sub-team independently reviewed the included articles, constructed codes from the surface meaning of the text and collated these into a code book, which was used to code and analyse the rest of the articles in an iterative process. New codes were associated with prior codes and concepts. An inductive approach allowed us to identify codes and themes from the raw data without using existing frameworks or preconceived notions as to how the data should be organized. The sub-team discussed their independent analyses in online and face-to-face meetings and used “negotiated consensual validation” to derive the final themes.

#### Directed content analysis

Three members of the research team independently employed Hsieh and Shannon’s approach [63] to directed content analysis. This involved “identifying and operationalizing a priori coding categories” by classifying text of similar meaning into categories drawn from prevailing theories. The research team first used deductive category application to extract codes and categories from Radha Krishna and Alsuwaigh [31]’s article, “Understanding the fluid nature of personhood – the Ring Theory of Personhood”. A code book was developed and individual findings were discussed through online and face-to-face meetings. Differences in codes were resolved until consensus was achieved on a final list of categories.

As part of the reiterative process within the SEBA methodology, the initial data was reviewed by the expert and research teams who determined that with current evolutions in concepts of MD extended to various aspects of moral principles and subject to individual, religious, cultural and societal considerations. As a result the expert team advised that the included articles be evaluated using categories drawn from Kuek et alChan, Chia [35]’s article entitled “Extending the Ring Theory of Personhood to the Care of Dying Patients in Intensive Care Units”, to determine the impact of dissonance or conflict between the values, beliefs and principles within individual rings and between the four rings.

### Stage 3 of SEBA: jigsaw perspective

The Jigsaw Perspective employs adopted Phases 4 to 6 of France, Uny [64]’s adaptation of Noblit, Hare [65]’s seven phases of meta-ethnography to view themes and categories identified in the Split Approach as pieces of a jigsaw puzzle. Here overlapping/complementary pieces are combined to create a bigger piece of the puzzle to create a wider/holistic view of the overlying data. This process would see themes and subthemes compared with the categories and subcategories identified. Similarities between the subthemes and subcategories are further compared with the codes contained to confirm the similarities and indeed if they are complementary in nature. If this is confirmed, then the subtheme and subcategory are combined to create a bigger piece of the jigsaw puzzle. Guided by the Jigsaw Perspective, these overlaps and similarities were combined to provide a holistic picture of available data on MD in medical students.

### Stage 4 of SEBA: funnelling process

A funnelling approach was adopted to streamline results from the three aspects of the Split Approach. It sees data compared and combined to reduce overlap and repetition whilst retaining a holistic perspective of the data.

#### Results

**Fig. 3:**
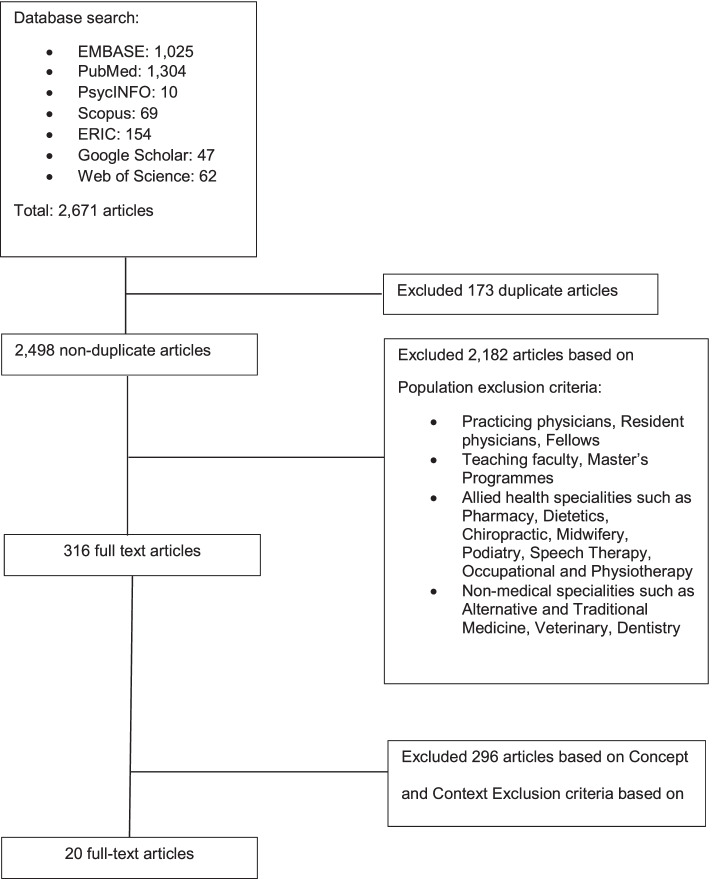
PRISMA flowchart

Two thousand six hundred seventy-one abstracts were identified from eight databases, 316 full text articles were reviewed, and 20 articles were included as shown in Fig. 3.

The themes identified were definitions, causes, impact, influencing factors, assessment, and interventions. The categories identified related to the four rings of the RToP, the Intra-ring conflicts (disharmony) and Inter-ring conflicts (dyssynchrony) (Table 2).

**Table 2:** Themes from Thematic Analysis (TA) and Direct Content Analysis (DCA)

Themes from Thematic Analysis (TA)		Themes/subthemes from Direct Content Analysis (DCA)		
Definition of MD	1. Dissonance between one’s ethical/moral beliefs and one’s action or behaviour 2. Constrained from doing the perceived ethically right thing	**Theme 1: Innate Ring**		
		Factors increasing levels of MD		a. Gender b. Religions, philosophies, and cultures c. Number of clinical years and experience
		**Theme 2: Individual Ring**		
		1. Precipitants for occurrence of moral distress		a. Observation/participation in self-perceived professional lapses b. Breaches in patient safety, confidentiality, consent c. Unpleasant experiences between healthcare providers and patient/patient’s family d. Students ‘ perception of ethical conflict due to moral stand
		2. Personal conception of morality		a. Personalized trade-offs in morally distressing situations determine a student’s choice of action b. Perspective on the morals of an ideal doctor c. Inadequate understanding of clinical ethics and its implications in medicine
		3. Coping with moral distress individually		a. Habituating to morally distressing scenarios b. Follow-up action by individuals to remedy moral distress c. Identifying role models to learn from
		4. Beliefs and perspectives that guard against moral distress		a. Doing what was requested will benefit patient b. Doing the act will aid learning c. Doing the act will help gain acceptance into medical fraternity d. Students are not directly responsible for the medical treatments decreases MD intensity
		5. Beliefs and perspectives that predispose to moral distress		a. Predisposition to moral distress when in conflict with personal beliefs of morality or medical care b. Perception of poor working environment c. Perception of power differential and its consequences d. Belief that patient is unable to make a sound medical decision and conflicts with appropriate medical care e. Failure to meet personal standard of morals and medical outcomes or treatment f. Underdeveloped, poor perspective of the role of medicine g. Self-perceived inadequacy to provide quality patient care h. Perceived societal constraints or inequalities that hamper access to treatment i. Self-perceived inability to cope with moral distress j. Poor professional identity
		6. The influence of emotions		a. Dual Process Theory - emotions influence beliefs or perspectives b. Discordant emotional responses from medical professionals c. Positive attitudes towards elderly patients
		7. Impact of moral distress on the individual		a. Burnout b. Wanting to quit the job c. Erosion of empathy d. Moral residue from previous MD e. Interest in geriatrics form increased MD occurrence f. Feelings of anger, sadness, anxiety
		**Theme 3: Societal Ring**		
		1. Victims of medical hierarchy		a. Difficulty in following instructions from senior doctors that they do not agree with b. Difficulty in speaking out against seniors due to vulnerable position in the hierarchy c. Afraid to clarify doubts about doctor’s actions d. Doubt arising from actions discordant from rest of medical term e. Inability to confront patient’s families about decisions that they disagree with f. Unclear role in the hierarchy / medical team
		2. Resource constraints compromising patient care		a. Insufficient time spent with patients b. Stretching of hospital resources
		3. Administrative impairment		a. Ineffective leadership and management b. Uncertainty regarding reporting protocol c. Inadequate knowledge of what is considered appropriate consent d. Medical curricula insufficient for moral growth
		4. Role of community in managing MD		a. Mentors enlightening medical students and developing their perspective b. Poor relationship with co-workers and poor sense of community c. Appropriate role modelling d. Negative role models e. Discussions and reflections to fabricate a ‘safe space’ for students to share and learn from one another f. Culture shift away from speaking up as an act of insubordination g. School responsibility to support students and intervene in morally distressing situations
		5. Societal pressures		a. The role of medical team to learn and gain skills and knowledge to become a doctor b. Implications of reporting an illegal medical conduct c. Students taught to prioritise patient autonomy d. Administration of medical therapy for safety of others e. Failure to care for less fortunate and at-risk f. Difficulty in ascertaining what is truly in patient’s best interests g. Inability to provide adequate treatment due to social problems
		6. Harmful societal effects of MD		a. Decreased manpower leading to resource constraints b. Negative impacts on patient care due to resource constraints, loss of empathy
		7. Personal involvement and choice		a. Face-to-face interpersonal situations
		**Theme 4: Intra-ring conflicts (Societal and Societal Ring)**		
		1. Disconnection of one’s own ideals and actual actions		a. Participating in professional lapses despite knowing that one should not b. Not being in control of patient’s outcome despite wanting to c. Balancing between prolonging patient’s life and preserving their quality of life d. Respecting patient autonomy despite knowing that action is not in the best interest of the patient e. Providing medications despite being aware of potential abuse or reliance f. Laughing off comments that one deems as inappropriate g. Wanting to do more for the patient but limited by resource constraints
		2. Respecting ethical principles while training to achieve competence		a. Practicing skills and procedures on patients without consent b. Practicing on more vulnerable groups of patients
		**Theme 5: Inter-ring conflicts**		
		1. Innate and Societal Ring conflict	a. Religion and the sanctity of life and the need to meeting clinical obligations	
Causes of MD	**Hierarchical structures** 3. Fear of repercussions 4. Fear of offending superiors 5. Fear of negative professional consequences **Healthcare systems** 1. Failure of healthcare system to give appropriate care 2. Lack of adequate follow-up, discharge plan 3. Sub-optimal care due to resource reduction 4. Level of care based on insurance 5. Lack of resources **Interactions of medical team with others** 1. Language barriers, poor communication 2. Lack of respect to other healthcare professionals 3. Lack of respect to patients **Ethical conflicts** 1. Patient autonomy and perceived beneficence to patient 2. Family’s wishes misaligned with assessed best interest of patient 3. Medical team’s actions and decisions and medical students’ perceived beneficence to patient 4. Patient autonomy and safety of others **Difference in values and beliefs** 2. Difference in beliefs from other HCP 3. Difference in ideals of profession and reality of role 4. Living up to expectations of others and core beliefs about professional identity **Self-doubt** A. Perceived lack of knowledge B. Perceived powerlessness due to lack of autonomy C. Lack of understanding of decision-making process D. Lower level of competency			
Factors affecting MD	**Risk factors** 1. Gender 2. Poor workplace relationships 3. Challenging, high-risk environments (ICU, ED) 4. Underdeveloped skills or professional identity 5. Interactions with vulnerable populations (elderly, children, disabled) 6. Institutional policies **Protective factors** 1. Frequency of exposure to distressing situations 2. Conducive health environments 3. Presence of training programmes 4. Guidance from positive role models 5. Good intra-HCP team relationships 6. Institutional policies			
Impact of MD	**Negative impacts to self** 1. Emotional and psychological distress (depression, anger, anxiety) 2. Erosion of empathy, emotional desensitization, and detachment 3. Feelings of guilt 4. Burnout, fatigue, and decreased well-being 5. Questioning of one’s moral integrity 6. Loss of passion and drive for medicine 7. Doubting one’s own career choices 8. Dropping out of medical school **Positive impact to self** 1. Develop new perspectives on purpose and meaning of medicine 2. Transformation of values, actions, or perception of actions **Impact on patient care** 1. Sub-optimal patient care, decreased quality of care 2. Withdrawal from direct patient care activities			
Tools to assess MD	Moral Distress Scale (MDS) Moral Distress Scale-Revised (MDS-R) Measure of Moral Distress – Healthcare Professionals (MMD-HP)			
Interventions to address MD	**Individual coping mechanisms** 1. Changing personal perceptions 2. Confronting the issue causing MD 3. Avoidance or inaction **Organisational interventions to increase communication** 1. Case-based small group discussion 2. Large group lecture 3. Reflective writings **Support and education** 1. Incorporating MD material into clinical teaching 2. Coinciding ethical teachings with clinical education 3. Training students on communication with colleagues and superiors 4. Educating mentors on how to deal with MD in medical students 5. Educating mentors with up-to-date professionalism policies Principles behind interventions 1. Incorporating case-based ethics education 2. “Speak up” culture 3. System oriented approaches to foster conducive environments 4. Early interventions to prevent build-up of moral residue Recommendations for the future 1. Medical training through curriculum changes 2. Institutional outreach to increase support 3. Changes in workplace culture			

The domains created by combining the themes and the subtheme and the categories and sub-categories are presented in Additional file 3: Appendix C to enhance reproducibility, accountability, and accountability of the Jigsaw Perspective.

The resultant domains were definitions of MD, sources of MD using RToP, recognition of MD and, interventions for MD.

### Domain 1: definition of MD

JametonJameton [66] attributes MD to feelings of powerlessness to do what healthcare professionals deemed was morally correct due to organizational restrictions [54]. This definition is widely adopted amongst the included articles [56–59] and echoed in the definitions in other papers [56, 57, 59, 62]. Recently the concept of MD has been expanded to include cognitive-emotional dissonance between one’s ethical/moral beliefs and actions or behaviour that one is compelled to perform [63–67]. MD can occur immediately or later and at an individual, team or system levels (Table 3).

**Table 3:** Existing definitions of Moral Distress amongst Medical Students

Title	Author	Definitions
Moral distress in the third year of medical school; a descriptive review of student case reflections	Lomis et al. 2009 [68]	Jameton’s definition.
Medical students’ experiences of moral distress: development of a web-based survey	Wiggleton et al. 2010 [69]	Jameton’s definition.
How Should Resident Physicians Respond to Patients’ Discomfort and Students’ Moral Distress When Learning Procedures in Academic Medical Settings?	Miller 2017 [70]	Jameton’s definition.
Moral distress in medical student reflective writing	Camp and Sadler 2019 [67]	Jameton’s definition.
Narrative, emotion and action: analysing ‘most memorable’ professionalism dilemmas	Rees et al. 2013 [71]	“Moral distress is when students feel unable to act in a manner consistent with their desire to do the ‘right’ thing.” (p. 93)
Antecedents and Consequences of Medical Students’ Moral Decision-Making during Professionalism Dilemmas	Monrouxe et al. 2017 [72]	“Moral distress, is emotional distress arising from the dissonance between one’s ethical/moral beliefs and one’s behaviour, which occurs when one’s actions are perceived as being limited by institutional constraints or unequal power relations. Moral distress can occur solely in the moment in which a person feels upset or uncomfortable (classified as mild distress) or continues for weeks or even months after an event (moderate distress). In extreme circumstances, distress is experienced many months or even years later (severe distress). Moral distress is different from other feelings.” (p. 568)
How Should Integrity Preservation and Professional Growth Be Balanced during Trainees’ Professionalization?	Weber and Gray 2017 [73]	Moral distress is “a negatively-valenced feeling state where one perceives a conflict between what one is expected to do and what morality requires.” (p. 545)
How Should Trainees Respond in Situations of Obstetric Violence?	Rubashkin and Minckas 2018 [74]	Moral distress is “the cognitive-emotional dissonance that arises when one feels compelled to act against one’s moral requirements.” (p. 240)
Joining the Club	Fuks 2018 [75]	The construct of moral distress is when “believes he or she knows the morally correct response to a situation but cannot act because of hierarchical or institutional constraints” (Lomis, Carpenter, and Miller 2009, p. 107).
Medical student reflections on geriatrics: Moral distress, empathy, ethics, and end of life	Camp 2018 [76]	Building on (Jameton, 1984)’s definition moral distress occurs when (1) A student described him- or herself doing or colluding with actions that the student believed were morally suspect or frankly immoral and (2) The student expressed that he or she was bothered by this to some degree. (p. 238)
Navigating Cognitive Dissonance: A Qualitative Content Analysis Exploring Medical Students’ Experiences of Moral Distress in the Emergency Department	Schrepel et al. 2019 [77]	Moral distress is defined as the negative feelings that arise when one knows the morally correct thing to do but they feel compelled to act in a way that contradicts with their values. (p. 332)
A systematic review of the causes, impact and response to moral distress among medical students	Glick 2019 [78]	Moral distress occurs when one is aware of the moral and ethical course of action yet is unable to perform it. (p. 1)
Medical students’ experiences of moral distress-a cross-sectional observational, web-based multicentre study	Dias 2020 [79]	Moral distress can be described as a psychological response to morally challenging situations, including moral conflict, dilemma, or uncertainty. Moral distress root causes can occur at patient, team or system levels.“ (p. 1)
Moral distress and burnout in caring for older adults during medical school training	Perni et al. 2020 [80]	1. Moral distress is a negative emotional state that results when a person feels inhibited from addressing a situation felt to be ethically problematic due to external constraints, including hierarchical or institutional constraints 2. We defined moral distress for respondents as “recognizing the situation to be ethically problematic and feeling inhibited from doing anything about it.” (p. 2)
Medical Students’ Experiences of Moral Distress in End-of-Life Care	Thurn and Anneser 2020 [81]	Moral distress occurs in situations in which a person recognizes a moral problem and has no doubts about the correct response but is constrained from acting on it or resolving it. (p. 116)
Ethikk First – extracurricular support for medical students and young physicians facing moral dilemmas in hospital routine	Kuhn et al. 2021 [82]	Such value conflicts cause moral stress, a term that was first introduced into the nursing sciences by the philosopher Andrew Jameton; however, it is now intensively being researched for various health professions. In a broad definition, it describes psychological reactions to moral challenges. (p. 2)

### Domain 2: sources of MD viewed through RToP lens

#### Innate ring

Female medical students are more likely to report MD than their male counterparts [58, 59, 65, 76, 81]. Aside from gender, religious, spiritual and cultural influences and clinical experience [56, 59, 63, 67, 69, 75, 78] are also sources of MD [62, 69, 81].

#### Individual ring

The medical students assessment of a situation, their moral standpoint [57–59, 62, 66, 67, 70, 81] and concepts of morality [59, 62, 67, 69, 78] can precipitate MD [58, 64–67, 81].

#### Relational ring

Social support from family and close friends protects against harmful effects of moral distress [68, 83].

#### Societal ring

Medical hierarchy also precipitates MD. This revolves around concerns that any dissent and or refusal to abide by the decisions of the senior physicians [56, 64, 66, 67, 69, 76, 81] would prevent the student from ‘fitting in’ [56, 57, 64–66]. This desire to ‘fit in’ also underlies resistance to question decisions and actions even when they may run contrary to professional obligations [56, 59, 62–67, 69, 76, 78–81]. MD is compounded by a [67, 68, 76, 79, 80]lack of clarity on the medical student’s role and influence on the team’s decisions and actions [56, 58, 66, 81].

Resource constraints and their implications on quality and access to healthcare also provoke MD [56, 57, 59, 64, 67, 76].

#### Conflicts

Conflicts are central to the concept of MD and when viewed through the lens of the RToP highlights intra-ring (disharmony) and inter-ring (dyssynchrony) conflict. Though they may occur concurrently, we highlight individual examples of disharmony and dyssynchrony.

#### Intra-ring conflicts

Dissonance between values and beliefs within a particular ring results in intra-ring conflicts or ‘disharmony’ [77, 84]. For example, ‘disharmony’ within the societal ring, may occur when a patient’s proposed actions run contrary to medical advice [59, 67, 74], or when medical students do not have an opportunity to meet their professional responsibilities such as reporting the abuse of the patient for fear of compromising the patient’s anonymity [76]. MD has also been reported when medical students feel conflicted about giving opioids to opioid dependent patients; witnessing patients undergoing unnecessarily ‘burdensome’ or even ‘futile’ treatment; or witnessing inadequate symptom control because the attending physician was not ‘comfortable’ to do so [56, 57, 59, 62–67, 69, 70, 74, 76, 78, 81].

Medical students also report MD when they struggle to maintain their professional responsibilities to the patient in the face of contradicting the decisions taken by the physicians [64, 78, 81] or when they feel conflicted when meeting their academic objectives [56, 70, 72, 74, 78] at the cost of what they conceive to be the patient’s choice to refuse [59, 62, 69], or performing tasks that the medical student does not feel confident nor equipped to carry out [62]. Miller et al. [62] describe a student “[beginning] to worry that if she performs the lumbar puncture, she would be putting her own interests as a student before those of her patient, who should always receive the best care possible”(p. 538).

#### Inter-ring conflicts

Tension between values, principles and beliefs between the rings or Inter-ring conflicts are termed dyssynchrony [77, 84]. Dyssynchrony is exemplified as [67–70, 72–80, 85, 86] medical students struggle with their desire to ‘fit in’ and compromise their ideals and beliefs [56, 65–67, 69, 70]. Similarly Dias [66], described medical students experiencing MD when “[participating] in care that [the medical student] does not agree with (abortion appointments)”(p. 6), highlighting the conflict between the Innate Ring with regards to religion and the sanctity of life and the need to meeting clinical obligations which would be part of the societal ring. [67, 69, 70, 73, 75, 84–86].

Administrative protocols [65, 76], healthcare inequality and resource constraints also increase the risk of dyssynchrony [56, 57, 59, 64, 65, 67].

### Domain 3: recognizing MD

[72] MD often manifests as anxiety, depression [81], a diminished sense of well-being and guilt and burnout and disillusionment and decreased empathy [58, 66, 74, 75]. In some cases, MD may even compromise patient care [56, 62, 67, 70].

MD is also detected through self-reporting via a variety of methods including self-administered surveys [58, 59, 65, 66] and or reflective essays [56, 64]. Yet self-reporting of MD may be compromised by concerns over the impact of such admissions upon career prospects [69, 85] and the lack of clear reporting processes [76].

There were no studies that reported the use of third-party assessment methods completed by tutors or peers that identify MD [67, 75, 78–81].

### Domain 4: interventions

If unaddressed, MD can precipitate disillusionment, self-doubt over a medical career [69] and dissatisfaction with the medical profession [63], burnout [58, 66] and exiting the medical school [56, 65].

Educational interventions to attenuate MD include increasing awareness of MD [57, 62, 65, 81], and ethical issues [65, 66] and enhancing communication skills [62, 64, 72, 81] and professionalism [72, 76, 77, 80].

Education interventions often take the form of case-based ethical discussions [72, 73], case-based small group discussions [68–70, 72, 73], large group lectures [72], reflective writing under peer and expert guidance [68, 73, 77, 81] and positive role modelling [68] that run longitudinally throughout the medical school training [81].

### Stage 5 of SEBA: analysis of evidence-based and non-data driven literature

Concerns over the quality of the data included from non-data-based articles (grey literature, opinion, perspectives, editorial, letters) and its potential impact upon the analysis of this review saw the themes drawn from evidenced-based publications were compared with those from non-data-based articles. This process found that the themes from both groups to be similar suggesting that information drawn from non-data based articles did not bias the analysis untowardly.

### Stage 6 of SEBA: discussion and synthesis of SSR in SEBA

[87, 88]The narrative produced by consolidating the tabulated summaries, themes and categories was guided by the Best Evidence Medical Education (BEME) Collaboration guide [87] and the STORIES (STructured apprOach to the Reporting In healthcare education of Evidence Synthesis) statement [88].

In addressing its primary and secondary research questions on what is known about MD, its causes, and the interventions to address MD in medical students, this SSR in SEBA highlights several key findings.

To begin, MD arises when conflicts that impact deeply held beliefs, values, and principles rooted in the medical student’s identity are not easily resolved. Through the lens of the RToP, such conflicts that underlie MD can be explained by the concepts of disharmony within and/or dyssynchrony between the rings. This process is further influenced by the medical student’s personal, existential, spiritual, familial, societal, cultural, and demographic factors, contextual considerations that influence the severity of these conflicts; their ability to process these conflicts; motivations; and the support structures available to them in addressing these conflicts.

These insights lend themselves to reports of MD amongst other healthcare professionals. For one, [1, 9, 10, 16, 17, 20, 89–108] nurses appear to have more intense episodes of MD due to feelings of powerlessness when faced with the medical hierarchy that belittles their input [1, 20, 97, 100, 103, 105, 108]. Therapists also report MD [108] due to their limited role in diagnosing and influencing care of medical ailments and the subsequent limitations in their roles in care and treatment determinations.

Accounts of MD in medical students and physicians were also largely similar, particularly amongst junior physicians [1, 19, 20, 93, 97, 100, 103, 105, 108]. Junior doctors, like their medical student counterparts, are more prone to MD due to their limited role in treatment decisions within the medical hierarchy [104, 109–113]. For both groups, there is an associated sense of helplessness that appears to recede with progress along the medical hierarchy.

Evidencing the notion that MD is a sociocultural construct informed by psychosocial, individual and contextual considerations, it is clear that assessment requires careful elucidation and a personalised and longitudinal approach. It is here that due consideration of the various values, beliefs and principles of each ring is key and the potential adaptation of the RToP as a tool to evaluate MD comes to light.

Concurrently treating MD requires a holistic and longitudinal perspective of MD and reiterates the need for active involvement of the medical schools in recognising, addressing, and attenuating the effects of MD and supporting medical students facing such distress. Aside from aiding in the diagnosis of MD and identifying medical students ‘at risk’ of MD, medical schools must provide robust and accessible means of support by training faculty to recognise and address MD [71, 85, 114, 115], and ensure the presence of a timely, personalised and ‘safe’ environment where medical students can discuss their concerns without fear of ‘reprisals’ upon their professional reputations and careers. In addition, there is a need to evaluate the hidden curriculum, and the introduction of initiatives such as speak-up culture [70, 79, 81]and anonymous reporting and feedback opportunities [67, 74] and supplementing positive role modelling and mentoring within the training program [68–70, 79], would be helpful. These considerations should also be accompanied by a clear delineation of the role and responsibilities of the medical student within the medical teams and the support available to them.

### Limitations

Even though we had the guidance of an expert team, the use of specific search terms and inclusion of only English language articles compounds the risk of omitting key articles and limiting the findings to North American and European settings. This may lead to the unintended exclusion of articles from other settings. As concepts of MD and personhood are sociocultural constructs, the omission of non-English articles may have significant ramifications on the applicability of these findings in Confucian-inspired societies [31, 116–119]. [31] Here relational autonomy, filial piety and family-centric associations play a critical role in self-concepts of identity and personhood and thus suggest that concepts of MD [120–125] in these settings may be different and not fully reflected by our findings.

## Conclusions

MD is a unique phenomenon determined by a medical student’s values, beliefs, goals, principles, perspectives, and contextual and psychoemotional considerations. In evidencing the complexity of this concept, the RToP has shown the potential to be adapted as a tool to evaluate MD holistically and in a socioculturally [119] appropriate manner. Such a tool could guide the support of medical students in need, and help design and oversee a safer learning and working environment for medical students. Concurrently with identity, contextual factors and psycho-emotional considerations constantly changing an RToP tool could also provide longitudinal follow up of medical students who have suffered MD.

Drawing on recent studies on longitudinal support and assessments of medical students the use of a tool to assess MD based on the RToP could be included within a medical student’s [126] to assess progress and direct support. As we look forward to engaging in this growing field, we are especially hopeful for greater understanding of the long-term effects of MD in various cultures and to evaluate the efficacy of support mechanisms for ‘at risk’ and ‘recovering’ medical students.

## Supplementary Information

**Additional file 1: Appendix A.** Pubmed Search Strategy. Search strategy employed as part of the systematic scoping review (SSR) process, Stage 1.

**Additional file 2: Appendix B.** Summary of included articles. Summaries of key points of articles included with MERSQI and COREQ.

**Additional file 3: Appendix C.** Summary of Direct-Content Analysis (DCA) Themes. Impact of Moral Distress according to the Innate, Individual, Relational and Societal Rings of Personhood.

## Data Availability

All data generated or analysed during this study are included in this published article and its supplementary information files.

## Abbreviations

MD: Moral Distress
SSR: Systematic Scoping Review
SEBA: Systematic Evidence Based Approach
RToP: Ring Theory of Personhood
YLLSoM: Yong Loo Lin School of Medicine
NCCS: National Cancer Centre Singapore
