# Supplementary material for: A systematic scoping review moral distress amongst medical students
Source: BMC Med Educ. 2022 Jun 17;22:466. doi: 10.1186/s12909-022-03515-3 (PMC9203147; doi:10.1186/s12909-022-03515-3)
Supplement: Supplementary file 1 — Additional file 1: Appendix A. Pubmed Search Strategy. Search strategy employed as part of the systematic scoping review (SSR) process, Stage 1. [file 12909_2022_3515_MOESM1_ESM.docx]

# **Appendix A**

## **Medline (via Pubmed) search strategy**

(("Physicians"[MeSH Terms] OR "students, medical"[MeSH Terms] OR "physician"[Title/Abstract] OR "Physicians"[Title/Abstract] OR "doctor"[Title/Abstract] OR "doctors"[Title/Abstract] OR "clinician"[Title/Abstract] OR "clinicians"[Title/Abstract] OR "resident"[Title/Abstract] OR "residents"[Title/Abstract] OR "general practitioner*"[Title/Abstract] OR (("medical"[Title/Abstract] OR "medicine"[Title/Abstract]) AND ("student"[Title/Abstract] OR "students"[Title/Abstract] OR "undergraduate*"[Title/Abstract] OR "postgraduate*"[Title/Abstract]))) AND ("moral obligations"[MeSH Terms] OR "ethics, medical"[MeSH Terms] OR "conscience"[MeSH Terms] OR (("moral"[Title/Abstract] OR "morals"[Title/Abstract] OR "morally"[Title/Abstract] OR "ethic"[Title/Abstract] OR "ethics"[Title/Abstract] OR "ethical"[Title/Abstract] OR "ethically"[Title/Abstract]) AND ("distress"[Title/Abstract] OR "distresses"[Title/Abstract] OR "distressing"[Title/Abstract] OR "distressed"[Title/Abstract] OR "dilemma"[Title/Abstract] OR "dilemmas"[Title/Abstract] OR "constraint"[Title/Abstract] OR "constraints"[Title/Abstract])))) AND ((english[Filter]) AND (1990:2021[pdat]))

## **Embase** ('physician'/exp OR 'medical student'/exp OR physician:ti,ab OR physicians:ti,ab OR doctor:ti,ab OR doctors:ti,ab OR clinician:ti,ab OR clinicians:ti,ab OR resident:ti,ab OR residents:ti,ab OR 'general practitioner*':ti,ab OR (((medical OR medicine) NEAR/4 (student OR students OR undergraduate* OR postgraduate*)):ti,ab)) AND ('moral obligations'/exp OR 'ethics, medical'/exp OR 'conscience'/exp OR ((moral OR morals OR morally OR ethic OR ethics OR ethical OR ethically) NEAR/4 (distress OR distresses OR distressing OR distressed OR dilemma OR dilemmas OR constraint OR constraints)):ti,ab) AND [embase]/lim NOT ([embase]/lim AND [medline]/lim) AND [1990-2021]/py AND [english]/lim

## **Psycinfo (via OVID)**

(exp Physicians/ or exp Medical Students/ or (physician or physicians or doctor or doctors or clinician or clinicians or resident or residents or "general practitioner*" or ((medical or medicine) adj5 (student or students or undergraduate* or postgraduate*))).ti,ab.) and (exp morality/ or exp bioethics/ or exp Conscience/ or ((moral or morals or morally or ethic or ethics or ethical or ethically) adj5 (distress or distresses or distressing or distressed or dilemma or dilemmas or constraint or constraints)).ti,ab.)

With year filter (from year 1990 to 2021), with language filter (English)

## **ERIC (via ProQuest)**

(MAINSUBJECT.EXACT.EXPLODE("Physicians") OR MAINSUBJECT.EXACT.EXPLODE("Medical Students") OR ti(physician OR physicians OR doctor OR doctors OR clinician OR clinicians OR resident OR residents OR "general practitioner*" OR ((medical OR medicine) NEAR/6 (student OR students OR undergraduate* OR postgraduate*))) OR ab(physician OR physicians OR doctor OR doctors OR clinician OR clinicians OR resident OR residents OR "general practitioner*" OR ((medical OR medicine) NEAR/6 (student OR students OR undergraduate* OR postgraduate*)))) AND (MAINSUBJECT.EXACT.EXPLODE("Moral Values") OR MAINSUBJECT.EXACT.EXPLODE("Ethics") OR ti((moral OR morals OR morally OR ethic OR ethics OR ethical OR ethically) NEAR/6 (distress OR distresses OR distressing OR distressed OR dilemma OR dilemmas OR constraint OR constraints)) OR ab((moral OR morals OR morally OR ethic OR ethics OR ethical OR ethically) NEAR/6 (distress OR distresses OR distressing OR distressed OR dilemma OR dilemmas OR constraint OR constraints)))

With year filter (from year 1990 to 2021), with language filter (English)

## **SCOPUS**

TITLE-ABS-KEY ( ( physician  OR  physicians  OR  doctor  OR  doctors  OR  clinician  OR  clinicians  OR  resident  OR  residents  OR  "general practitioner"  OR  "general practitioners"  OR  ( ( medical  OR  medicine )  W/5  ( student  OR  students  OR  undergraduate*  OR  postgraduate* ) ) )  /10W  ( ( moral  OR  morals  OR  morally  OR  ethic  OR  ethics  OR  ethical  OR  ethically )  W/5  ( distress  OR  distresses  OR  distressing  OR  distressed  OR  dilemma  OR  dilemmas  OR  constraint  OR  constraints ) ) )  AND  (LIMIT-TO (PUBYEAR , 2021) OR  LIMIT-TO ( PUBYEAR ,  2020 )  OR  LIMIT-TO ( PUBYEAR ,  2019 )  OR  LIMIT-TO ( PUBYEAR ,  2018 )  OR  LIMIT-TO ( PUBYEAR ,  2017 )  OR  LIMIT-TO ( PUBYEAR ,  2016 )  OR  LIMIT-TO ( PUBYEAR ,  2015 )  OR  LIMIT-TO ( PUBYEAR ,  2014 )  OR  LIMIT-TO ( PUBYEAR ,  2013 )  OR  LIMIT-TO ( PUBYEAR ,  2012 )  OR  LIMIT-TO ( PUBYEAR ,  2011 )  OR  LIMIT-TO ( PUBYEAR ,  2010 )  OR  LIMIT-TO ( PUBYEAR ,  2009 )  OR  LIMIT-TO ( PUBYEAR ,  2008 )  OR  LIMIT-TO ( PUBYEAR ,  2007 )  OR  LIMIT-TO ( PUBYEAR ,  2006 )  OR  LIMIT-TO ( PUBYEAR ,  2005 )  OR  LIMIT-TO ( PUBYEAR ,  2004 )  OR  LIMIT-TO ( PUBYEAR ,  2003 )  OR  LIMIT-TO ( PUBYEAR ,  2002 )  OR  LIMIT-TO ( PUBYEAR ,  2001 )  OR  LIMIT-TO ( PUBYEAR ,  2000 )  OR  LIMIT-TO ( PUBYEAR ,  1999 )  OR  LIMIT-TO ( PUBYEAR ,  1998 )  OR  LIMIT-TO ( PUBYEAR ,  1997 )  OR  LIMIT-TO ( PUBYEAR ,  1996 )  OR  LIMIT-TO ( PUBYEAR ,  1995 )  OR  LIMIT-TO ( PUBYEAR ,  1994 )  OR  LIMIT-TO ( PUBYEAR ,  1993 )  OR  LIMIT-TO ( PUBYEAR ,  1992 )  OR  LIMIT-TO ( PUBYEAR ,  1991 )  OR  LIMIT-TO ( PUBYEAR ,  1990 ) )  AND  ( LIMIT-TO ( LANGUAGE ,  "English" ) )

## **Web Of Science**

TS=( ( physician OR physicians OR doctor OR doctors OR clinician OR clinicians OR resident OR residents OR "general practitioner" OR "general practitioners" OR ( ( medical OR medicine ) NEAR/5 ( student OR students OR undergraduate* OR postgraduate* ) ) ) NEAR/10 ( ( moral OR morals OR morally OR ethic OR ethics OR ethical OR ethically ) NEAR/5 ( distress OR distresses OR distressing OR distressed OR dilemma OR dilemmas OR constraint OR constraints ) ) )

With year filter (from year 1990 to 2021), with language filter (English)

## Google Scholar

allintitle: doctor OR doctors OR physician OR physicians OR clinician OR clinicians OR resident OR residents OR “general practitioners” OR “medical students” OR “medical student” "moral distress" OR "ethical dilemma" OR "ethical dilemmas"

With year filter (from year 1990 to 2021)

## **Open grey**

lang:"en" AND ((physician OR physicians OR doctor OR doctors OR clinician OR clinicians OR resident OR residents OR "general practitioner” OR “general practitioners” OR (medical OR medicine) AND (students OR students OR undergraduate OR undergraduates OR postgraduate OR postgraduates)) AND ((ethic OR ethics) OR distress)) AND (year:(2021) OR year:(2020) OR year:(2019) OR year:(2018) OR year:(2017) OR year:(2016) OR year:(2015) OR year:(2014) OR year:(2013) OR year:(2012) OR year:(2011) OR year:(2010) OR year:(2009) OR year:(2008) OR year:(2007) OR year:(2006) OR year:(2005) OR year:(2004) OR year:(2003) OR year:(2002) OR year:(2001) OR year:(2000) OR year:(1999) OR year:(1998) OR year:(1997) OR year:(1996) OR year:(1995) OR year:(1994) OR year:(1993) OR year:(1992) OR year:(1991) OR year:(1990))
