# Supplementary material for: A systematic scoping review moral distress amongst medical students
Source: BMC Med Educ. 2022 Jun 17;22:466. doi: 10.1186/s12909-022-03515-3 (PMC9203147; doi:10.1186/s12909-022-03515-3)
Supplement: Supplementary file 2 — Additional file 2: Appendix B. Summary of included articles. Summaries of key points of articles included with MERSQI and COREQ. [file 12909_2022_3515_MOESM2_ESM.docx]

| Author/year | Article title | Type of study | MERSQI | COREQ | Study aim | Methodology | Key findings | Proposed solutions/ conclusions |
| --- | --- | --- | --- | --- | --- | --- | --- | --- |
| Tamara Thurn, Dipl-Psych, and Johanna Anneser, MD  2020 | Medical Students' Experiences of Moral Distress in End-of-Life Care | Qualitative | NA | 1-4 | To assess the frequency and intensity of medical students’ moral distress occurring in end-of-life care. | We developed a questionnaire describing 10 potentially morally distressing scenarios in end-of-life care. The questionnaire was distributed to all fourth-year students of a German medical school. We asked students (1) if they had ever witnessed the described scenarios and (2) to rate the extent (numeric rating scale 0–4) of moral distress for each situation. | Of 340 students, 217 (64%) completed the survey. On average, students had experienced 2.51 morally distressing situations (standard deviation =–2.23). The majority of students (N= 163, 75%) had experienced at least one morally distressing situation. Providing futile care with the basic intention to make money was the item with the highest levels of experienced distress (2.88 – 1.05), witnessed by 54 (25%) participants. Twenty-five students (12%) reported that they had thought about dropping out of medical school or choosing a nonclinical specialty because of moral distress | Medical students experience moral distress regularly and most frequently in scenarios of futile care. This may be an underestimated factor for medical school attrition. Interventions should identify the sources of moral distress and empower students to address their moral concerns. |
| Dias, Mónica Patrícia Silva  2020 | Medical students' experiences of moral distress-a cross-sectional observational, web-based multicentre study | Quantitative | 10 | NA | The aims of this study are to translate and culturally adapt the “Measure of Moral Distress – Healthcare Professionals (MMD-HP)” questionnaire; to collect additional relevant data using other questions; and to explore the frequency and intensity of moral distress occurring among medical students in seven participating Portuguese medical schools. | This was a multi-phase, multi-centre, cross-sectional study. First, we translated and culturally adapted the “Measure of Moral Distress – Healthcare Professionals (MMD-HP)” into Portuguese, following the internationally accepted “COnsensus-based Standards for the selection of health Measurement INstruments (COSMIN)”, insuring it was suitable for medical students to use. Then, we conducted a web-based survey, following the “Checklist for Reporting Results of Internet E-Surveys (CHERRIES)” guidelines. Students from the seven Portuguese medical schools included in the study were asked to rate 27 potentially morally distressing situations on two dimensions: frequency and intensity, both in a 5 numeric rating scale with 0 representing no distress. The survey also included 5 multiple choice questions related to the topic and sociodemographic items. Free, informed consent was obtained by each potential participant in the form of clicking a square next to the statement declaring the aim of the study, which also included their right to withdrawal at any point with no consequences. The study was approved by all seven medical schools’ ethics committees included in this work. | Our study showed that most Portuguese medical students had already experienced morally distressing situations as early as first years of clinical practice. These experiences may promote medical school dropouts with its discussed consequences and may affect the way future healthcare professionals deal with clinical and ethical challenging situations, contributing to moral residue and crescendo effect of moral distress. | Our data suggests moral distress is a common phenomenon among medical students and its experience shows a cumulative effect over time. Medical schools might adapt their curricula in order to address this phenomenon and mitigate its effects as early as first clinical years of medical education. |
| Subha Perni , Lauren R. Pollack , Wendy C. Gonzalez , Elizabeth Dzeng and Matthew R. Baldwin  2020 | Moral distress and burnout in caring for older adults during medical school training | Mixed methods | 11 | 1-5 | Moral distress is a reason for burnout in healthcare professionals, but the clinical settings in which moral distress is most often experienced by medical students, and whether moral distress is associated with burnout and career choices in medical students is unknown. We assessed moral distress in medical students while caring for older patients, and examined associations with burnout and interest in geriatrics. | A cross-sectional survey study of second-, third-, and fourth-year medical students at an American medical school. The survey described 12 potentially morally distressing clinical scenarios involving older adult patients. Students reported if they encountered each scenario, and whether they experienced moral distress, graded on a 1–10 scale. We conducted a principal axis factor analysis to assess the dimensionality of the survey scenarios. A composite moral distress score was calculated as the sum of moral distress scores across all 12 scenarios. Burnout was assessed using the Maslach Abbreviated Burnout Inventory, and interest in geriatrics was rated on a 7-point Likert scale. | Medical students experience moral distress in caring for older adults, and higher levels of moral distress are asso- ciated with burnout. Students’ responses suggest that a focused thread of didactic sessions in inpatient geriatric care and debriefing sessions with peers and faculty that are integrated into inpatient clinical clerkships on medi- cine, neurology, and surgery might mitigate the effects of moral distress and subsequent burnout. | Moral distress is highly prevalent among medical students while caring for older patients, and associated with burnout. Incorporating geriatrics education and debriefing sessions into inpatient clerkships could alleviate medical student moral distress and burnout. |
| Catherine Wiggleton, MD, Emil Petrusa, PhD, Kim Loomis, MD, John Tarpley, MD, Margaret Tarpley, Mary Lou O’Gorman, MDiv, and Bonnie Miller, MD  2010 | Medical students' experiences of moral distress: development of a web-based survey | Quantitative | 9 | NA | To develop an instrument for measuring moral distress in medical students, measuring the prevalence of moral distress in a cohort of students, and identifying the situations most likely to cause it. Moral distress, defined as the negative feelings that arise when one knows the morally correct thing to do but cannot act because of constraints or hierarchies, has been documented in nurses but has not been measured in medical students. | The authors constructed a survey consisting of 55 items describing potentially distressing situations. Responders rated the frequency of these situations and the intensity of distress that they caused. The survey was administered to 106 fourth-year medical students during a three-week period in 2007; the response rate was 60%. | Each of the situations was experienced by at least some of the 64 respondents, and each created some degree of moral distress. On average, students witnessed almost one-half of the situations at least once, and more than one-third of the situations caused mild-to-moderate distress. The survey measured individual distress (Cronbach alpha = 0.95), which varied among the students. Whereas women witnessed potentially distressing situations significantly more frequently than did men (P = .04), men tended to become more distressed by each event witnessed (P = .057). | Medical students frequently experience moral distress. Our survey can be used to measure aspects of the learning environment as well as individual responses to the environment. The variation found among student responses warrants further investigation to determine whether students at either extreme of moral distress are at risk of burnout or erosion of professionalism. |
| Mary Camp & John Sadler  2019 | Moral distress in medical student reflective writing | qualitative | NA | 1-2 | Moral distress occurs when one identifies an ethically appropriate course of action but cannot carry it out. In this conceptualization, medical students may be particularly vulnerable to moral distress, but the literature on moral distress in medical trainees remains sparse. | Using content analysis of 802 reflective essays written by third-year medical students, the authors analyzed for the presence of moral distress and other ethical themes. The authors then used chi-squared analysis to determine which ethical themes were statistically associated with moral distress. | Two hundred and seventy-four (34%) of the essays included student descriptions of moral distress. The most frequent theme in the moral distress essays was  “role of the medical student” in the training hierarchy, and this reached a statistically significant association with moral distress (x^2^ = 15.19, p<0.001). Statistically significant associations (p<0.05) were also found with moral distress and themes related to an “ethical disagreement with supervisor,”“insensitive care,”“disputes,”“abuse,”“poverty,”“medical errors,” and  “transplant ethics.” Essays discussing the “doctor–patient relationship” or observations of a “job well done” were statistically less likely to involve moral distress. | Moral distress is a common occurrence in medical students, particularly related to medical students’ role in the training hierarchy or other difficult interpersonal and clinical interactions. In our sample, moral distress was described less often in the presence of positive role models. |
| Kimberly D. Lomis, M.D.a,*, Robert O. Carpenter, M.D.a, Bonnie M. Miller, M.D.b  2009 | Moral distress in the third year of medical school; a descriptive review of student case reflections | Qualitative | NA | 107-108 | Medical students may find certain clinical experiences particularly difficult. Moral distress occurs when a trainee sees a situation or behavior as undesirable, but, because of a position in the hierarchy, declines to address the problem. To prompt our students to reflect on such experiences, students are required to submit a brief case description and are assigned to mentor groups to discuss cases. | After exemption from our Institutional Review Board, a database of student submissions was de-identified. A total of 192 case descriptions were analyzed by a single reviewer to identify recurrent themes. Submissions were categorized in a binary fashion as higher or lower levels of distress. Frequency and correlation with levels of distress were assessed for each theme. | Sixty-seven percent of the submissions were classified as higher distress. Seven major  themes were identified, the most common being problems of communication (n = 179). Those students taking action correlated to lower distress. | Our review shows that specific situations can be expected to generate moral distress in trainees. Addressing such distress may support the ongoing professional growth of trainees. |
| Caitlin Schrepel, MD, Joshua Jauregui, MD , Alisha Brown, MD, Jamie Shandro, MD, MPH, and Jared Strote, MD, MS  2019 | Navigating Cognitive Dissonance: A Qualitative Content Analysis Exploring Medical Students' Experiences of Moral Distress in the Emergency Department | qualitative | NA | 331-338 | As undergraduate medical students are acculturated into clinical practice, they develop a set of refined professional values that impact their decision making. We aimed to use students’ reflective narratives on ethical dilemmas to identify how students experience moral distress while working in the emergency department (ED) to better understand how to support them in the development of their own agency to act ethically. | Students rotating in our emergency medicine clerkship are required to submit an essay describing an ethical dilemma they encountered. We selected a random sample of these reflective pieces from the 2015 and 2016 academic years and used an exploratory qualitative thematic analytic approach to identify frequently recurring themes. This process was continued until thematic sufficiency was reached. | Two-hundred essays were coded, and seven unique themes were identified. The moral distress students described in reflective writing narratives stemmed from patient-provider discord, uncertainty, and social injustices. In each case, students were expressing the cognitive dissonance they experienced as they began to reconcile the difference between their perceptions of optimal patient care and the actual care delivered to the patient. | Understanding medical students’ cognitive dissonance in the ED will help educators support their students as they negotiate the differences between preferences and principles while being acculturated into clinical practice. Future work should develop specific interventions to promote educator understanding of learners’ moral distress and to develop novel models of support for learners. |
| Mary E. Camp, Haekyung Jeon-Slaughter, Anne E Johnson & John Z Sadler  2017 | Medical student reflections on geriatrics: Moral distress, empathy, ethics and end of life | qualitative | NA | 1-18 | Medical students’ early clinical encounters may influence their perceptions of geriatrics. This study examines reflective essays written by 3rd year medical students on required clinical rotations. | Using content analysis, the authors analyzed the essays’ thematic content. The authors then used chi-square analysis to compare themes with geriatric patients (age 60+) to themes with other age groups. | One hundred twenty out of 802 essays described a geriatric patient. The most common geriatric themes were (1) death and dying, (2) decision making, (3) meaningful physician-patient interactions, (4) quality of care, and (5) professional development. Geriatric essays were more likely to discuss death/dying and risk-benefit themes and less likely to discuss abuse. Geriatric essays were more likely to describe students' moral distress. Geriatric essays with moral distress were more likely to include empathy themes compared to geriatric essays without moral distress. | Geriatric patients may pose unique ethical challenges for early clinical students. |
| Tamara Thurn, Dipl-Psych, and Johanna Anneser, MD  2019 | Medical Students' Experiences of Moral Distress in End-of-Life Care | quantitative | 8.5 | NA | To assess the frequency and intensity of medical students’ moral distress occurring in end-of-life care. | Design: We developed a questionnaire describing 10 potentially morally distressing scenarios in end-of-life care.  Setting: The questionnaire was distributed to all fourth-year students of a German medical school.  Measurements: We asked students (1) if they had ever witnessed the described scenarios and (2) to rate the extent (numeric rating scale 0–4) of moral distress for each situation. | Of 340 students, 217 (64%) completed the survey. On average, students had experienced 2.51 morally distressing situations (standard deviation = ±2.23). The majority of students (N = 163, 75%) had experienced at least one morally distressing situation. Providing futile care with the basic intention to make money was the item with the highest levels of experienced distress (2.88 ± 1.05), witnessed by 54 (25%) participants. Twenty-five students (12%) reported that they had thought about dropping out of medical school or choosing a nonclinical specialty because of moral distress. | Medical students experience moral distress regularly and most frequently in scenarios of futile care. This may be an underestimated factor for medical school attrition. Interventions should identify the sources of moral distress and empower students to address their moral concerns. |
| Charlotte E Rees, Lynn V Monrouxe & Laura A McDonald  2013 | Narrative, emotion and action: analysing ‘most memorable’ professionalism dilemmas | Qualitative and Quantitative | 8 | 80-93 | Although previous studies have explored medical learners’ ‘most memorable’ experiences, these have typically focused on patient deaths or mistakes. Drawing on multiple theoretical perspectives to understand the interplay between narrative, emotion and action, this paper aims to explore the whats and hows of written narratives of most memorable professionalism dilemmas: what types of dilemma are most memorable? When and where do they take place? How do students act? What characteristics relate to these dilemmas? How are dilemmas narrated? | A total of 680 students from 29 of 32 UK medical schools provided a written narrative of their most memorable dilemma as part of their responses to an online questionnaire exploring the impact of professionalism dilemmas on moral distress. We employed quantitative thematic and discourse analysis of all narratives using Linguistic Inquiry Word Count software (LIWC) and conducted a narrative analysis of one exemplar. | The most common themes across all narratives concerned dilemmas that related to issues of patient care with reference to the actions of health care professionals or students, student abuse, and consent and intimate examination. A total of 41.1% of experiences had occurred over 6 months previously and 80.1% had taken place in hospital settings. Overall, 54.9% of narrators reported having done something in the face of their dilemma, although only 13.2% described taking obvious or direct action. Numerous characteristics were related to most memorable dilemmas (e.g. narratives citing intimate examinations were more likely to take place in surgical settings). A total of 92.6% of narratives included negative emotion talk and numerous significant relationships emerged between types of emotion talk and most memorable dilemmas (e.g. more anger talk in abuse narratives). Our narrative analysis of one exemplar illustrates the richness of emotion talk and more subtle devices to establish emotional tone | Findings extend previous research into issues related to professionalism by exploring relationships between narrative, emotion and action in the context of written narratives of most memorable dilemmas. We encourage medical educators to help students construct coherent and emotionally integrated narratives to make sense of negative professionalism dilemmas. |
| Renato Soleiman Franco, Camila Ament Giuliani Franco, Solena Ziemer Kusma, Milton Severo & Maria Amélia Ferreira  2017 | To participate or not participate in unprofessional behavior - is that the question? | Quantitative | 11 | NA | Medical education provides students with abundant learning opportunities, each of which is embodied with messages concerning what is expected from students. This paper analyses students’ exposure to instances of unprofessional behavior, investigating whether they judge such behavior to be unprofessional and whether they also participate in unprofessional behavior | The survey developed in the Pritzker School of Medicine at the University of Chicago was the basis of this questionnaire that was answered by 276 students from two medical schools in Brazil and Portugal. | Unprofessional behavior was observed frequently by students in both universities, and the mean participation rates were similar (26% and 27%). Forty-five percent of students’ participation in unprofessional behavior was explained by academic year, prior observation, and judgment. | Proper discussion of unprofessional behavior should foster a broad debate to encourage empowered students, faculties, and physicians to co-create a more professional environment for patient care. |
| Kimberly E. Kopecky, MD, Tiffany J. Zens, MD, Pasithorn A. Suwanabol, MD, MS, and  Margaret L. Schwarze, MD, MPP  2018 | Third-year medical students’ reactions to surgical patients in pain: doubt, distress, and depersonalisation | Qualitative | NA | 720-724 | The objective of this study was to characterize the experiences of medical students as they encounter pain,  suffering, and the emotional experiences of doctoring. | We used qualitative analysis to explore the content of 341 essays written by third-year medical students who  described their experiences with surgical patients in pain. We used an inductive process to develop a coding taxonomy and then  characterized the content of these essays related to empathy, patient-clinician interaction, and descriptions of clinical norms. | Students found it difficult to reconcile patient suffering with the therapeutic objective of treatment. They feared  an empathic response to pain might compromise the fortitude and efficiency required to be a doctor and they pursued  strategies to distance themselves from these feelings. Students described tension around prescription of pain medications and  worried about the side effects of medications used to treat pain. Students felt disillusioned when operations caused suffering  without therapeutic benefit or were associated with unexpected complications. Although patients had expressed a desire for  intervention, students worried that the burdens of treatment and long-term consequences were beyond patient imagination. | These observations about patient-doctor relationships suggest that there is a larger problem among clinicians  relating to patient distress and personal processing of the emotional nature of patient care. Efforts to address this problem will  require explicit instruction in skills to develop a personal strategy for managing the emotionally challenging aspects of clinical  work. |
| Eva Kuhn,  Laura Lunden, Penelope Moysich,  Kai Rogge,  Marijke Roscher,  Lotta Caning,  Annette Rogge  2021 | Ethik First – extracurricular support for medical students and young physicians facing moral dilemmas in hospital routine | Quantitative | 8 | NA | The multimodal concept is based on three pillars:  In monthly principle-based case conferences, participants practice  ethical reflection and moral judgment primarily on the basis of concrete  cases introduced by them using the methods of problem-based learning  and consideration-based deliberation. If participants do not bring forth Intensive Care Medicine, Kiel,  a case, they discuss ethical aspects of current political relevance. Germany  Moreover, there is an annual public speaker event. | Since the project began in 2017, ~20 students and interns  have taken part in Ethik First one or more times. In a web-based interim  evaluation (N=13), all respondents fully agreed that they considered  the format helpful for dealing with ethical questions at the clinic. They  rated the relevance for their later profession as high. There is evidence  for support in moral dilemma situations. | The first evaluation results of the voluntary extracurricular  offer show the acceptance of the selected format, which goes beyond  pure teaching in its conception in that it addresses moral stress as well  and strengthens the participants’ individual resilience. | Ethik First reinforces the role of ethical aspects in the  training of (prospective) doctors and focuses on reflecting on cases they  have experienced firsthand.  We formulate a desideratum for appropriate advanced training concepts  both in medical studies and in advanced medical training such that the  training and development of comparable projects at medical faculties  and at medical associations with student participation can be discussed |
| Lynn Monrouxe, PhD, Malissa Shaw, MSc, PhD, and Charlotte Rees, MEd, PhD 2017 | Antecedents and Consequences of Medical Students' Moral Decision Making during Professionalism Dilemmas | Tertiary | NA | NA | We suggest how medical educators can support students' understandings of ethical dilemmas and facilitate their habits of enacting professionalism: by modeling appropriate resistance behaviors. | By highlighting the concepts of professionalism dilemmas and moral decision making, we examine various types of dilemmas encountered by students, how they respond to those dilemmas, and any resultant moral distress they experience. Finally, we offer suggestions for how medical educators, working at the student, faculty, and organizational levels, can reduce or prevent students’ professionalism lapses in the face of ethical dilemmas, thereby reducing their moral distress. |  |  |
| Eli Weber, PhD, MA, and Sharon Gray, BSN, PHN, RN 2017 | How Should Integrity Preservation and Professional Growth Be Balanced during Trainees' Professionalization? | Tertiary | NA | NA |  |  |  | Thus, it is important for health professions training programs to incorporate case-based ethics education sessions into their structure to help identify and alleviate trainees’ moral distress, provide ethics education, and create a “safe space” for trainees to talk openly about moral concerns related to clinical practice. |
| Bonnie M. Miller, MD, MMHC 2017 | How Should Resident Physicians Respond to Patients' Discomfort and Students' Moral Distress When Learning Procedures in Academic Medical Settings? | Tertiary | NA | NA | This article argues that trainees should have the opportunities to practice procedures and difficult conversations in simulated settings and that institutions should support a culture of “speaking up” to ensure patients’ and learners’ safety. |  |  | This article argues that trainees should have the opportunities to practice procedures and difficult conversations in simulated settings and that institutions should support a culture of “speaking up” to ensure patients’ and learners’ safety. |
| Nicholas Rubashkin, and Nicole Minckas 2018 | How Should Trainees Respond in Situations of Obstetric Violence? | Tertiary | NA | NA |  |  | We show that the student’s situation can be understood as one of moral distress and argue that, in this specific instance, it would be appropriate for the student to intervene by providing supportive care to the patient. | However, we suggest that medical schools have an obligation to better prepare students for rotations conducted abroad. |
| Dominic E. Sanford  David A. Fleming  2010 | We meant no harm, yet we made a mistake; why not apologize for it? A student's view | Tertiary | NA | NA | This essay explores the unique perspective of medical students regarding the ethical challenges of providing full disclosure to patients and their families when medical mistakes are made, especially when such mistakes lead to tragic outcomes. |  | Ethical principles cannot be abandoned in fear of adverse evaluation or failure to conform. Healthcare workers have an obligation to address mistakes made around the time of a patient’s death with the patient’s family. This responsibility trumps any selfish desire to avoid unpleasant feelings of guilt or regret. | Such events often bring closure to already anguished relatives and spouses, and may help to facilitate the grieving process. This includes pressing forward the need to apologize to patients and/or their families when mistakes are made and when decisions are made that lead to poor outcomes for the patient, even when benevolently intended. |
| Abraham Fuks  2018 | Joining the club | Tertiary | NA | NA |  | This essay is grounded in an exegesis of narratives proffered by senior medical students during a selective course on the language of medicine. | These stories reveal the affinity of students for their patients and their sensitivity to inappropriate behaviors by physicians and other caregivers. By contrast, observing role models who enact respect for patients reinforces the natural regard of students towards patients as persons and engenders respect of students for their teachers. Students grapple with moral dilemmas that contribute to cognitive dissonance in their lived realities. They arrive at medical school with powerful and sometimes clear images of their calling as physicians yet encounter antithetical role models whose behaviors contradict their own evolving identities. | In consequence, medical students may wonder whether entry to the club they are eager to join entails abandoning both their patients and their ideals. |
| Danish Zaidi  Jacob A. Blythe  Benjamin W. Frush  Jay R. Malone  2020 | Clerkship ethics: unique ethical challenges for physicians-in-training | Tertiary | NA | NA |  |  | Three ethical conflicts in particular are paradigmatic of what we define as “clerkship ethics.” First, a distinction that differentiates the clerkship student from the practicing physician involves the student’s principal role as a learner. Second, evaluative scrutiny during clinical clerkships often forces medical students to balance doing what is morally fitting against the perceived expectations of the medical teams in which they work. Third and finally, a deeply entrenched culture of medical hierarchy presents a particular challenge to innovation and improvement in ethics education during the clerkship years. | Students regard faculty as exemplars, but are not provided with the tools to assess when technical medical competence is not matched by moral competence; moreover, these faculty are unlikely to have experienced the ethics education in which students are asked to demonstrate mastery. |
| Susan B. Glick; Jacob Schulman; Max Harris; Daniel Pohlman. Rush Medical College, Chicago, IL  2019 | A systematic review of the causes, impact and response to moral distress among medical students | Abstract | NA | NA | To prevent, diagnose and treat moral distress among medical students requires an understanding of the causes and impact of moral distress, as well as the response to it at the personal, professional and institutional levels. | With the assistance of a medical librarian, we conducted a systematic review of the English language literature to assess studies that described moral distress among medical students. We searched OVID, CINAHL, PsycINFO, and ERIC from their inception through October 2018. We searched the reference sections of relevant review articles as well as all included studies for additional manuscripts. Eligible studies had to represent an original study; present data about the causes, impact, or response to moral distress; measure and report outcomes in medical students; and report findings in English. Titles and abstracts were reviewed. Articles not recommended for exclusion were reviewed in full. Following full text review, those articles that did not meet all four inclusion criteria were excluded. | The search yielded 63 articles. Following title, abstract and full text review, 6 studies were included. Five addressed situations that result in moral distress; two addressed its impact. No studies addressed the response to moral distress. Due to data heterogeneity, narrative synthesis was performed. Studies differed in their conclusions about which situations resulted in the greatest moral distress, including those with immediate potential to cause harm; covert, status-related and verbal abuse; communication issues, intra-team conflicts, resource allocation, and access to care; and experiences with geriatric patients. Role modeling was inversely correlated with student distress, as was action-taking. One study found repeated exposure to moral distress can result in either emotional desensitization or in worsening distress. Studies differed in their conclusions about whether moral distress disproportionately affected women or men. | Little is known about the situations that result in moral distress among medical students and its impact. The literature is silent on the response to medical students' moral distress. Deepening our understanding of the causes, impact and response to moral distress among medical students is imperative to promote student wellness. |
