# Supplementary material for: A systematic scoping review moral distress amongst medical students
Source: BMC Med Educ. 2022 Jun 17;22:466. doi: 10.1186/s12909-022-03515-3 (PMC9203147; doi:10.1186/s12909-022-03515-3)
Supplement: Supplementary file 3 — Additional file 3: Appendix C. Summary of Direct-Content Analysis (DCA) Themes. Impact of Moral Distress according to the Innate, Individual, Relational and Societal Rings of Personhood. [file 12909_2022_3515_MOESM3_ESM.docx]

|  | Predispose to MD | | Attenuate MD | |
| --- | --- | --- | --- | --- |
| Innate Ring | Gender, with females experiencing greater amounts of MD | (Wiggleton, Petrusa et al. 2010, Monrouxe, Shaw et al. 2017, Rubashkin and Minckas 2018, Perni, Pollack et al. 2020, Thurn and Anneser 2020) |  |  |
|  | Relative clinical inexperience | (Perni, Pollack et al. 2020) |  |  |
| Individual Ring | Observation/participation in self-perceived professional lapses (breaches in patient safety, confidentiality, consent) | Monrouxe, Shaw et al. (2017)  (Schrepel, Jauregui et al. 2019)  (Thurn and Anneser 2020)  (Dias 2020)  (Perni, Pollack et al. 2020)  (Fuks 2018)  (Wiggleton, Petrusa et al. 2010)  (Camp and Sadler 2019)  (Lomis, Carpenter et al. 2009) | Habituating to morally distressing scenarios | (Miller 2017) |
|  | Professionalism dilemmas that may see values, beliefs and principles in conflict | Monrouxe, Shaw et al. (2017)  (Miller 2017)  (Schrepel, Jauregui et al. 2019)  (Camp, Jeon-Slaughter et al. 2018)  (Thurn and Anneser 2020)  (Dias 2020)  (Perni, Pollack et al. 2020)  (Fuks 2018)  (Wiggleton, Petrusa et al. 2010)  (Camp and Sadler 2019)  (Lomis, Carpenter et al. 2009) | Identifying role models to learn from  Role models helping students handle experiences with death well | (Camp, Jeon-Slaughter et al. 2018)  (Glick, Schulman et al. 2019)  (Lomis, Carpenter et al. 2009) |
|  | Inadequate understanding of morality and its implications in medicine (and thus the need to teach ethics/PIF) | (Weber and Gray 2017)  (Rubashkin and Minckas 2018) (Monrouxe, Shaw et al. 2017)  (Dias 2020) | Doing what was requested will benefit patient | Monrouxe, Shaw et al. (2017) |
|  | Variable understanding of the morals of an ideal doctor/ what kind of doctor they want to be | (Weber and Gray 2017)  (Miller 2017)  (Schrepel, Jauregui et al. 2019)  (Dias 2020) | Doing the act will aid learning | (Monrouxe, Shaw et al. 2017)  (Fuks 2018) |
|  | poor working conditions  -high stress  - hierarchical practice | (Weber and Gray 2017)  (Miller 2017)  (Rubashkin and Minckas 2018)  (Dias 2020)  (Wiggleton, Petrusa et al. 2010)  (Camp and Sadler 2019)  (Lomis, Carpenter et al. 2009) | Doing the act will help gain acceptance into medical fraternity | (Monrouxe, Shaw et al. 2017) |
|  | Poor patient care  -disrespectful behaviour  -causing harm  -providing misleading information  -ignoring patient’s wishes  -breaching moral and professional practice standards  - usurping patient choice  - Paternalistic practice  - willingness to place own interests and personal gain over the interests if the patient | (Monrouxe, Shaw et al. 2017)  (Miller 2017)  (Schrepel, Jauregui et al. 2019)  (Camp, Jeon-Slaughter et al. 2018)  (Thurn and Anneser 2020)  (Dias 2020)  (Perni, Pollack et al. 2020)  (Fuks 2018)  (Camp and Sadler 2019)  (Lomis, Carpenter et al. 2009) | Students do not have professional responsibilities | (Dias 2020) |
|  | Discordant emotional responses by medical professionals | (Camp, Jeon-Slaughter et al. 2018) |  |  |
|  | Burnout | (Monrouxe, Shaw et al. 2017)  (Miller 2017)  (Perni, Pollack et al. 2020) |  |  |
|  | Wanting to quit | (Monrouxe, Shaw et al. 2017)  (Perni, Pollack et al. 2020)  (Fuks 2018)  (Glick, Schulman et al. 2019) |  |  |
|  | Erosion of empathy | (Miller 2017)  (Fuks 2018) |  |  |
|  | Feelings of anger, sadness, anxiety | (Fuks 2018)  (Glick, Schulman et al. 2019) |  |  |
| Societal ring | medical hierarchy  -Preforming tasks that run contrary to own beliefs and concepts of professionalism  - cannot whistle blow  -compromising the learning opportunities of medical students  - forced to ignore poor behaviour | (Lomis, Carpenter et al. 2009, Miller 2017, Monrouxe, Shaw et al. 2017, Weber and Gray 2017, Fuks 2018, Rubashkin and Minckas 2018, Camp and Sadler 2019, Dias 2020, Thurn and Anneser 2020) | Mentors | (Miller 2017, Weber and Gray 2017) |
|  | Inability to confront patient’s families about decisions that they do not agree with | (Wiggleton, Petrusa et al. 2010, Camp, Jeon-Slaughter et al. 2018) (Camp, Jeon-Slaughter et al. 2018, Perni, Pollack et al. 2020) | sense of community | (Lomis, Carpenter et al. 2009, Wiggleton, Petrusa et al. 2010, Schrepel, Jauregui et al. 2019) |
|  | Resource limitations  -time constraints  -limited resources  -inadequate hospital support/delays  - poor management of resources  -poor access or treatment of vulnerable population | Thurn and Anneser 2020)(Lomis, Carpenter et al. 2009, Camp, Jeon-Slaughter et al. 2018, Camp and Sadler 2019, Glick, Schulman et al. 2019, Schrepel, Jauregui et al. 2019, Dias 2020) | a ‘safe space’ for students to share and learn from one another | (Weber and Gray 2017, Rubashkin and Minckas 2018) |
|  |  |  | Program support and oversight | (Lomis, Carpenter et al. 2009, Rubashkin and Minckas 2018, Schrepel, Jauregui et al. 2019) |

| Ring | Disharmony | |
| --- | --- | --- |
| Societal | Prioritising training over patient interests | (Miller 2017, Schrepel, Jauregui et al. 2019) |
| Individual | Knowingly participating in actions that breach standards of practice | (Lomis, Carpenter et al. 2009, Monrouxe, Shaw et al. 2017, Weber and Gray 2017) |
| Dyssynchrony | | |
| Individual  v societal | Provision of treatment and care that may be suboptimal as a result of patient’s choices | (Lomis, Carpenter et al. 2009, Wiggleton, Petrusa et al. 2010, Camp, Jeon-Slaughter et al. 2018, Schrepel, Jauregui et al. 2019, Perni, Pollack et al. 2020, Thurn and Anneser 2020) (Wiggleton, Petrusa et al. 2010, Rubashkin and Minckas 2018) |
| Innate vs Societal | Provision of legalised abortions when they run against religious and moral values | (Dias 2020) |

Camp, M. and J. Sadler (2019). "Moral distress in medical student reflective writing." AJOB Empir Bioeth **10**(1): 70-78.

Camp, M. E., H. Jeon-Slaughter, A. E. Johnson and J. Z. Sadler (2018). "Medical student reflections on geriatrics: Moral distress, empathy, ethics and end of life." Gerontol Geriatr Educ **39**(2): 235-248.

Dias, M. P. S. (2020). "Medical students' experiences of moral distress-a cross-sectional observational, web-based multicentre study."

Fuks, A. (2018). "Joining the Club." Perspect Biol Med **61**(2): 279-293.

Glick, S. B., J. Schulman, M. Harris and D. Pohlman (2019). "A systematic review of the causes, impact and response to moral distress among medical students." Journal of General Internal Medicine **34**(2): S119-S120.

Lomis, K. D., R. O. Carpenter and B. M. Miller (2009). "Moral distress in the third year of medical school; a descriptive review of student case reflections." Am J Surg **197**(1): 107-112.

Miller, B. M. (2017). "How Should Resident Physicians Respond to Patients' Discomfort and Students' Moral Distress When Learning Procedures in Academic Medical Settings?" AMA J Ethics **19**(6): 537-543.

Monrouxe, L., M. Shaw and C. Rees (2017). "Antecedents and Consequences of Medical Students' Moral Decision Making during Professionalism Dilemmas." AMA J Ethics **19**(6): 568-577.

Perni, S., L. R. Pollack, W. C. Gonzalez, E. Dzeng and M. R. Baldwin (2020). "Moral distress and burnout in caring for older adults during medical school training." BMC Med Educ **20**(1): 84.

Rubashkin, N. and N. Minckas (2018). "How Should Trainees Respond in Situations of Obstetric Violence?" AMA J Ethics **20**(1): 238-246.

Schrepel, C., J. Jauregui, A. Brown, J. Shandro and J. Strote (2019). "Navigating Cognitive Dissonance: A Qualitative Content Analysis Exploring Medical Students' Experiences of Moral Distress in the Emergency Department." AEM Educ Train **3**(4): 331-339.

Thurn, T. and J. Anneser (2020). "Medical Students' Experiences of Moral Distress in End-of-Life Care." J Palliat Med **23**(1): 116-120.

Weber, E. and S. Gray (2017). "How Should Integrity Preservation and Professional Growth Be Balanced during Trainees' Professionalization?" AMA J Ethics **19**(6): 544-549.

Wiggleton, C., E. Petrusa, K. Loomis, J. Tarpley, M. Tarpley, M. L. O'Gorman and B. Miller (2010). "Medical students' experiences of moral distress: development of a web-based survey." Acad Med **85**(1): 111-117.
